# Risk Factors for Influenza among Health Care Workers during 2009 Pandemic, Toronto, Ontario, Canada

**DOI:** 10.3201/eid1904.111812

**Published:** 2013-04

**Authors:** Stefan P. Kuster, Brenda L. Coleman, Janet Raboud, Shelly McNeil, Gaston De Serres, Jonathan Gubbay, Todd Hatchette, Kevin C. Katz, Mark Loeb, Donald Low, Tony Mazzulli, Andrew Simor, Allison J. McGeer, on behalf of the Working Adult Influenza Cohort Study Group

**Affiliations:** Mount Sinai Hospital, Toronto, Ontario, Canada (S.P. Kuster, B.L. Coleman, D. Low, T. Mazzulli, A.J. McGeer);; University of Toronto, Toronto (B.L. Coleman, J. Raboud, K.C. Katz, D. Low, T. Mazzulli, A. Simor, A.J. McGeer);; Dalhousie University, Halifax, Nova Scotia, Canada (S. McNeil, T. Hatchette);; Laval University Québec, Québec City, Québec, Canada (G. De Serres); Ontario Agency for Health Protection and Promotion, Toronto (J. Gubbay, D. Low);; North York General Hospital, Toronto (K.C. Katz);; McMaster University, Hamilton, Ontario, Canada (M. Loeb);; Sunnybrook Health Sciences Centre, Toronto (A. Simor).

**Keywords:** Influenza, virus, viruses, pandemic, health care worker, risk, hand hygiene, transmission, respiratory, ventilation, aerosol, Canada

## Abstract

Influenza was associated with household exposure, aerosol-generating procedures, and lower adherence to hand hygiene recommendations.

## Medscape CME Activity

Medscape, LLC is pleased to provide online continuing medical education (CME) for this journal article, allowing clinicians the opportunity to earn CME credit. 

This activity has been planned and implemented in accordance with the Essential Areas and policies of the Accreditation Council for Continuing Medical Education through the joint sponsorship of Medscape, LLC and Emerging Infectious Diseases. Medscape, LLC is accredited by the ACCME to provide continuing medical education for physicians.

Medscape, LLC designates this Journal-based CME activity for a maximum of 1 *AMA PRA Category 1 Credit(s)*^TM^. Physicians should claim only the credit commensurate with the extent of their participation in the activity.

All other clinicians completing this activity will be issued a certificate of participation. To participate in this journal CME activity: (1) review the learning objectives and author disclosures; (2) study the education content; (3) take the post-test with a 70% minimum passing score and complete the evaluation at www.medscape.org/journal/eid; (4) view/print certificate.

**Release date: March 18, 2013; Expiration date: March 18, 2014**

## Learning Objectives

Upon completion of this activity, participants will be able to:

- Define an influenza pandemic in this study
- Report signs and symptoms of influenza
- Determine whether the risk of influenza was higher in health care workers (HCWs) than in non-HCWs
- Report risk factors for influenza among HCWs

## CME Editor

**Jean Michaels Jones,** Technical Writer/Editor, Emerging Infectious Diseases. *Disclosure: Jean Michaels Jones has disclosed no relevant financial relationships*.

## CME Author

**Hien Nghiem, MD, **freelance writer, Medscape, LLC. *Disclosure: Hien Nghiem, MD, has disclosed no relevant financial relationships*.

## Authors

*Disclosures:* ***Stefan Kuster, MD; Brenda Coleman, PhD; Janet Raboud, PhD; Shelly McNeil, MD; Jonathan Gubbay, MBBS; Kevin Katz, MD; Mark Loeb, MD; Donald Low, MD; Andrew Simor, MD;*** *and* ***Allison McGeer, MD,*** *have disclosed no relevant financial relationships.* ***Gaston De Serres, MD, PhD,*** *has disclosed the following relevant financial relationships: served as an advisor for GlaxoSmithKline; received grants for clinical research from GlaxoSmithKline and* ***Sanofi Pasteur*** *.* ***Todd Hatchette, MD,*** *has disclosed the following relevant financial relationships: received grants for clinical research from GlaxoSmithKline.* ***Tony Mazzulli, MD,*** *has disclosed the following relevant financial relationships: served as a speaker for Merck.*
