# Supplementary material for: Risk Factors for Influenza among Health Care Workers during 2009 Pandemic, Toronto, Ontario, Canada
Source: Emerg Infect Dis. 2013 Apr;19(4):606–15. doi: 10.3201/eid1904.111812 (PMC3647716; doi:10.3201/eid1904.111812)
Supplement: Technical Appendix — Selection and recruitment of non–health care workers enrolled in Influenza Cohort Study, Toronto, Ontario, Canada [file 11-1812-Techapp-s1.pdf]

Publisher: CDC; Journal: Emerging Infectious Diseases
Article Type: Research; Volume: 19; Issue: 4; Year: 2013; Article ID: 11-1812
DOI: 10.3201/eid1904.111812; TOC Head: Research
Article DOI: http://dx.doi.org//10.3201/eid1904.121812

Risk Factors for Influenza among Health Care Workers during 2009 Pandemic, Canada

Technical Appendix

Selection and Recruitment of Non–Health Care Workers

Non–health care workers (non-HCWs) were primarily recruited from 20 participating office-based non–health care employers in downtown Toronto. At participating employers, flyers, intranet postings, email notices, information sessions, and information tables were used to recruit participants.

Non-HCWs were not eligible if they worked in any health care setting or if they had daily occupational face-to-face contact with numerous children or adults (e.g., teachers, daycare workers, sales clerks). There were no exclusions based on nonoccupational contact with children or adults. Although we excluded adults with occupational face-to-fact contact with numerous children or adults, the combination of heterogeneity of occupations, and lack of evidence regarding risk factors for influenza in healthy working adults meant that it was not possible to select workers such that their occupational risk was randomly assorted and representative of the population of workers who are not HCWs. Because, in our view, the epidemiology of influenza and a single previous study suggested that working in health care would not pose a risk for infection by influenza, we attempted to bias our non-HCWs in favor of identifying an occupational risk associated with health care, which would strengthen the conclusion if, indeed, we did not identify a risk associated with health care. We also deliberately selected employers in downtown Toronto, where our hospitals were located, such that exposure to public transit would be expected to be similar between groups because of some evidence for other respiratory diseases that frequent use of public transit increases the risk for infection.

It is, of course, possible that unmeasured confounders exist: adults who work in office setting that do not expose them to large numbers of children and adults may have systematically

different non-occupational risks of influenza than other types of workers. However, we think it unlikely that people whose occupation exposes them to numerous children or adults would systematically take particular care to avoid exposure to infection in other areas of their life.

Sample Size Calculation

Before the pandemic, we had developed the protocol as a study of seasonal influenza over 3 seasons. The sample size for this study required 225 non-HCW seasons and 1420 HCW seasons, in order to be able to identify and label as statistically significant an odds ratio of 2.0 (with an average infection rate of 5% in HCWs) for occupational risk associated with health care compared to office work. The ratio of HCW to non-HCW participants was selected to allow us to detect a 3-fold increase in risk in HCWs undertaking “high-risk” activities compared with other HCWs.

It is obviously difficult to perform a sample size calculation in the setting of a pandemic. We made a decision to attempt to recruit the aforementioned number of participants to a pandemic study, recognizing that the timing of both vaccine availability and influenza activity were unpredictable. By the onset of the second wave of the pandemic, we had enrolled ≈50% of our target. We continued to enroll; however, the third wave of the pandemic was very small in Ontario, and the complexities of attempting to assess risk over time in the second wave were such that we elected to analyze the data only for participants exposed during the entire second wave of the pandemic. Therefore, our study may not have been adequately powered to exclude a difference in influenza risk between HCWs and non-HCWs.
