# Supplementary material for: Risk Factors for Influenza among Health Care Workers during 2009 Pandemic, Toronto, Ontario, Canada
Source: Emerg Infect Dis. 2013 Apr;19(4):606–15. doi: 10.3201/eid1904.111812 (PMC3647716; doi:10.3201/eid1904.111812)
Supplement: Related texts

# Research article: Risk Factors for Influenza among Health Care Workers during 2009 Pandemic, Toronto, Ontario, Canada

**Authors:** Stefan P. Kuster, Brenda L. Coleman, Janet Raboud, Shelly McNeil, Gaston De Serres, Jonathan Gubbay, Todd Hatchette, Kevin C. Katz, Mark Loeb, Donald Low, Tony Mazzulli, Andrew Simor, Allison J. McGeer, on behalf of the Working Adult Influenza Cohort Study Group

**Affiliations:** Mount Sinai Hospital, Toronto, Ontario, Canada (S.P. Kuster, B.L. Coleman, D. Low, T. Mazzulli, A.J. McGeer);; University of Toronto, Toronto (B.L. Coleman, J. Raboud, K.C. Katz, D. Low, T. Mazzulli, A. Simor, A.J. McGeer);; Dalhousie University, Halifax, Nova Scotia, Canada (S. McNeil, T. Hatchette);; Laval University Québec, Québec City, Québec, Canada (G. De Serres); Ontario Agency for Health Protection and Promotion, Toronto (J. Gubbay, D. Low);; North York General Hospital, Toronto (K.C. Katz);; McMaster University, Hamilton, Ontario, Canada (M. Loeb);; Sunnybrook Health Sciences Centre, Toronto (A. Simor).

## Abstract

This prospective cohort study, performed during the 2009 influenza A(H1N1) pandemic, was aimed to determine whether adults working in acute care hospitals were at higher risk than other working adults for influenza and to assess risk factors for influenza among health care workers (HCWs). We assessed the risk for influenza among 563 HCWs and 169 non-HCWs using PCR to test nasal swab samples collected during acute respiratory illness; results for 13 (2.2%) HCWs and 7 (4.1%) non-HCWs were positive for influenza. Influenza infection was associated with contact with family members who had acute respiratory illnesses (adjusted odds ratio [AOR]: 6.9, 95% CI 2.2–21.8); performing aerosol-generating medical procedures (AOR 2.0, 95% CI 1.1–3.5); and low self-reported adherence to hand hygiene recommendations (AOR 0.9, 95% CI 0.7–1.0). Contact with persons with acute respiratory illness, rather than workplace, was associated with influenza infection. Adherence to infection control recommendations may prevent influenza among HCWs.

The numerous outbreaks of influenza described in acute care hospitals indicate that influenza transmission in this setting is of major concern (*1**–**3*). Nonetheless, it remains unclear whether health care workers (HCWs) are at higher risk for infection than are adults working in nonclinical settings (non-HCWs). Vaccination recommendations for HCWs are intended primarily to protect patients from hospital-acquired influenza and influenza-associated death (*4*,*5*). Although working in hospitals has been proposed as a risk factor for influenza (*6*), findings that support that working in health care settings poses an occupational risk (*7*), or that performing particular activities or working in specific health care disciplines are associated with an increased risk for influenza infection, are sparse.

Better understanding of risk factors for infection among HCWs would support decision-making regarding priorities for seasonal influenza vaccination, antiviral treatment or prophylaxis programs, implementation of other measures to reduce influenza transmission in hospitals, and planning for pandemics. Therefore, we aimed to assess risk factors for influenza among HCWs and to determine whether, during the first 2 waves of influenza A(H1N1)pdm09, HCWs working in acute care hospitals were at higher risk than non-HCWs for symptomatic influenza.

## Materials and Methods

### Participants and Setting

The Influenza Cohort Study, initiated by the Working Adult Influenza Cohort Study Group, a research team based in Toronto, Ontario, Canada, was started in May 2009. The purpose of the study was to examine incidence, clinical features, and epidemiology of infection caused by A(H1N1)pdm09 among HCWs and other working adults in Canada. For this analysis, participants were enrolled during May 29–September 27, 2009. Participants were eligible if they were 18–75 years of age and either worked >8 hours per week in 1 of 5 acute care hospitals (HCW) or in an office-based setting in Toronto (non-HCW). Non-HCWs were intended to provide a sample of working adults at low occupational risk for influenza, so as to bias the study toward the ability to identify an occupational risk in health care. Details of the recruitment of these control participants are included in the Technical Appendix. The study was approved by the Research Ethics Boards of all participating hospitals and universities and by the human resources departments of participating employers.

Upon enrollment, participants received a collection kit, an illustrated guide, and instruction from a nurse for mid-turbinate nasal swab sample self-collection. They also completed a Web-based questionnaire detailing influenza vaccination history, underlying medical conditions, demographic data, potential work- or school-related risk factors for respiratory virus infection, and potential community risk factors. Blood samples were taken from consenting participants at enrollment and again in April or May of 2010. 

Participants were asked to complete weekly Web-based diaries from enrollment until March 31, 2010, detailing respiratory symptoms and acute respiratory illness (ARI) or febrile illnesses and documenting time-dependent risk factors (e.g., contact with persons with ARI symptoms). Per the study protocol, if any signs or symptoms suggestive of an ARI developed, participants provided a self-collected mid-turbinate nasal swab sample as soon as possible after onset to be tested for influenza by using PCR. ARI was defined as 1) fever without another obvious source; or 2) new symptoms, including >2 of the following: runny or stuffy nose, sneezing, sore or scratchy throat, hoarseness, or cough; or 3) one local (runny/stuffy nose, sneezing, sore/scratchy throat, hoarseness, or cough) and 1 systemic symptom (fever, malaise, myalgia, headache, or fatigue).

Participants whose specimens tested positive for influenza were offered treatment in accordance with public health recommendations (*8*). All participants with undetermined A(H1N1)pdm09 vaccine status as of March 31, 2010, were contacted again to confirm whether they had received it and, if so, when. For logistical reasons, participants with unconfirmed 2009–2010 seasonal influenza vaccine status could not be contacted again; instead, these participants were assumed not to have received it. In Canada, vaccine for A(H1N1)pdm09 became available for HCWs and patients at high risk for complications of influenza during calendar week 43 (starting October 25, 2009) and was available for healthy adults during calendar week 47 (starting November 22).

### Definitions

For this study, HCW were defined as persons working in an acute care hospital. A non-HCW was defined as a person working in an office-type environment not associated with the provision of health care. The first and second waves of the influenza pandemic in Ontario were defined as the periods for which the weekly proportion of respiratory specimens that were positive for A(H1N1)pdm09 was >5%, as reported by the Ontario Agency for Health Protection and Promotion. Similarly, seasonal influenza waves were defined as periods for which >5% of weekly specimens tested positive for seasonal influenza. By this definition, the first pandemic wave occurred during calendar weeks 21–31 of 2009 (May 17–August 8); the second wave occurred during calendar weeks 39–48 (September 27–December 5). Peak weeks were defined as weeks during which positivity rates were >15% and comprised calendar weeks 21–27 (May 17–July 11) during wave 1 and calendar weeks 41–46 (October 11–November 21) during wave 2. As expected, few cases of seasonal influenza were identified during the study period.

Aerosol-generating medical procedures were defined as any of the following: administration of nebulized therapy or humidified oxygen at >40%, use of bag-valve mask, manual ventilation, noninvasive ventilation, open airway suctioning, bronchoscopy or other upper airway endoscopy, tracheostomy, endotracheal intubation, cardiopulmonary resuscitation, oscillatory ventilation, or any procedure that involved manipulation of open ventilator tubing in a mechanically ventilated patient or sputum induction or other deliberate induction of coughing.

Adherence to hand hygiene and facial protection recommendations was defined as the self-reported proportion of situations during which hand hygiene and facial protection were performed according to infection control recommendations (*9*). Symptomatic influenza infection was defined as influenza-positive PCR results for a participant-collected mid-turbinate nasal swab sample.

### Antibody Assays and Interpretation

Serum specimens were extracted from blood samples and 1 mL aliquots frozen at −70°C. Aliquots were tested by hemagglutination-inhibition (HAI) assay to determine antibody titers against the A(H1N1)pdm09 strain (A/California/07/2009-like) and the 2008–09 seasonal A(H1N1) strain (A/Brisbane/59/07) to identify potential cross-reactivity by using a protocol adapted from World Health Organization methods (*10*). Two HAI assays were performed per aliquot by using 0.5% turkey erythrocytes and 4 hemagglutination units per 25 µL of virus. For discordant pairs, the higher of the 2 geometric mean titers was used. Serum specimens were tested at the Queen Elizabeth II Health Sciences Centre, Halifax, Nova Scotia, Canada. Seroprotection was defined as having HAI antibody titers of >40. Seroconversion was specifically defined as a prevaccination HAI titer of <10 and a postvaccination titer of >40 or a 4-fold change in titers for participants with a prevaccination titer of >10 (*11*,*12*).

### Data Management and Statistical Analyses

Data were entered online by the participants, then cleaned and manually inspected for errors and outlying values. Differences in group proportions were assessed by the χ^2^ or Fisher exact test, as appropriate, and differences in means (for normally distributed data, on the basis of the Shapiro-Wilk test for normality) and medians (for non-normally distributed data) were calculated by using Student *t* test and Wilcoxon rank-sum test, respectively.

The analysis for the primary objective (i.e., to determine whether the risk for laboratory-confirmed symptomatic influenza was higher in HCWs than in non-HCWs) included all participants who were enrolled by the start of the second wave of the 2009 H1N1 influenza pandemic (calendar week 39, starting September 27, 2009). Multivariable generalized estimating equation logistic regression analysis was used to determine adjusted odds ratios with 2-sided 95% CIs for constant and time-dependent risk factors for symptomatic influenza infection on the basis of information from baseline questionnaires and weekly diaries. Model construction was performed on the basis of the method proposed by Harrell (*13*) including A(H1N1)pdm09 vaccination status and changing risk for influenza infection over time (community influenza activity). Our a priori approaches to adjust for changing risk for influenza infection over time were to 1) adjust for weekly percentage of specimens positive for influenza reported to the Ontario Agency for Health Protection and Promotion (continuous variable) and 2) adjust for peak weeks (defined as weeks during which >15% of specimens were positive for influenza; [dichotomous variable]). Vaccine failure among participants was defined as acquiring A(H1N1)pdm09 infection after receipt of A(H1N1)pdm09 vaccine >7 days before symptom onset. Participants who acquired A(H1N1)pdm09 within 7 days after vaccination were considered not fully protected. To evaluate the validity of this assumption, we performed sensitivity analyses by calculating lags of 0 days and 14 days, respectively. The same criteria were used in the analysis of the secondary objective (i.e., to determine risk factors for laboratory-confirmed symptomatic influenza among HCWs). The models with the lowest quasi-likelihood under the independence model criterion were preferred.

Data were analyzed in SAS, version 9.1 for PC (SAS Institute, Cary, NC, USA). We considered p values <0.05 as statistically significant.

### Sample Size

This study was initiated at the onset of the 2009 influenza pandemic; because the expected incidence of infection was unknown, a formal sample size was not established. Details of the sample size estimate for the planned seasonal study can be found in the online Technical Appendix.

## Results

### Study Population, Symptomatic Influenza Case-patients and Community Influenza Activity

The first participant was enrolled in the study on May 28, 2009 (calendar week 21). By October 11 (calendar week 41), at the start of the second wave of the pandemic, 732 participants were enrolled in the Influenza Cohort Study: 563 (76.9%) were HCWs who worked in 1 of 5 community and teaching acute care hospitals in the Toronto area and 169 (23.1%) were non-HCWs who worked in an office environment not associated with the provision of health care (Table 1; Figure). Of the 2 cohorts, HCWs were younger and were more likely to have been vaccinated against seasonal and pandemic influenza, to work with children, to have children <5 years of age in their households, and to use public transportation >8 times per week. Of 422 HCWs who were vaccinated against A(H1N1)pdm09, 403 (95.5%) received vaccine within 2 weeks after its availability; of 61 non-HCWs, 28 (45.9%) were vaccinated during the same time period (p<0.001).

**Table 1:** Characteristics of study participants in the Influenza Cohort Study performed during 2009 pandemic, Toronto, Ontario, Canada*

Characteristic	Participant values by cohort, n = 732		
	No. HCWs in acute care facilities, n = 563	No. non-HCWs in office settings, n = 169	p value
Mean age, y (± SD)	42.2 (11.3)	45.4 (10.8)	0.001
Female sex	478/563 (84.9)	133/169 (78.7)	0.06
Recipient of vaccine			
A(H1N1)pdm09 vaccine†	469/554 (84.7)	66/165 (40.0)	<0.001
Seasonal influenza vaccine 2009–10	226/563 (40.1)	41/169 (24.3)	<0.001
Seasonal influenza vaccine 2008–09	407/552 (73.7)	76/164 (46.3)	<0.001
Underlying health conditions			
Asthma	55/555 (9.9)	14/167 (8.4)	0.56
Diabetes mellitus	22/555 (4.0)	5/165 (3.0)	0.58
Allergies to airborne irritants	235/509 (46.2)	60/155 (38.7)	0.10
Current smoker or smoker in household	73/551 (13.3)	27/163 (16.6)	0.28
Potential exposure conditions			
Hand-to-face habits	275/557 (49.4)	76/168 (45.2)	0.35
Wearing of prescription eyeglasses	386/561 (68.8)	119/168 (70.8)	0.62
Reusable water bottle use >1/week	232/556 (41.7)	81/169 (47.9)	0.15
Public transit: >8 trips per week	196/558 (35.1)	45/168 (26.8)	0.04
Group gathering attendance	524/563 (93.1)	150/167 (89.8)	0.17
Face-to-face contacts/d, median (IQR)	10 (5, 20)	10 (5, 20)	0.94
>1 person/bedroom in household	192/551 (34.9)	51/166 (30.7)	0.33
Children in workplace	62/558 (11.1)	9/167 (5.4)	0.03
Child <5 y in household	77/563 (13.7)	10/169 (5.9)	0.006
Child <18 y in household	212/563 (37.7)	59/169 (34.9)	0.52
Child in household attends day care	77/555 (13.9)	16/164 (9.8)	0.17

*Data are no./total (%) unless otherwise specified. HCW, health care worker; A(H1N1)pdm09, pandemic influenza A(H1N1) 2009 virus; IQR, interquartile range. †Participants who had acquired A(H1N1)pdm09 <7 d after vaccination were considered unprotected.

**Figure:** Flowchart of 732 persons enrolled in the Influenza Cohort Study, Toronto, Ontario, Canada

A total of 334 (45.6%) study participants submitted 436 nasal swab samples. More than half (52.1%) of these samples were collected on the day of symptom onset (day 1), 19.4% on day 2, 9.9% on day 3, and 12.1% on or after day 4. Among the 20 (4.6%) specimens yielding influenza, 12 (60.0%) were collected on day 1, four (20.0%) on day 2, three (15.0%) on day 3, and one (5.0%) on day 4 of illness. Thirteen (2.2%) of 563 HCWs and 7 (4.1%) of 169 non-HCWs submitted samples that tested positive for influenza. A(H1N1)pdm09 was detected in 19 (95%) of the 20 positive participants: 1 case during each of calendar weeks 24, 25, 31, 39, 40, and 47; two cases during each of calendar weeks 42, 44, and 45; and 7 cases during calendar week 43. Thus, 16 of 19 cases occurred during weeks of peak A(H1N1)pdm09 activity. Seasonal influenza A(H3N2) virus was isolated in a sample from 1 participant during calendar week 43.

### Risk Factors for Symptomatic Influenza Infection

The probability of symptomatic influenza infection did not differ between HCWs and non-HCWs (p = 0.28) (Table 2). Study participants who had a child <18 years of age living in the household (36.2% of influenza negative/untested participants vs. 65.0% of influenza positive participants; p = 0.009), a child who attended day care living in the same household (12.5% vs. 30.0%; p = 0.03), and who were not vaccinated against A(H1N1)pdm09 >7 days before onset of infection (76.3% vs. 10.0%; p<0.001) were more likely to have respiratory illness with positive test results for influenza.

**Table 2:** Results of influenza virus testing of nasal swab specimens from 732 study participants in the Influenza Cohort Study performed during 2009 pandemic, Toronto, Ontario, Canada*

Characteristic	Influenza test status		
	No. negative or not ill, n = 712†	No. positive, n = 20‡	p value
Mean age, y (± SD)	42.9 (11.3)	43.9 (9.3)	0.69
Female sex	595/712 (83.6)	16/20 (80.0)	0.76
Worker in acute care hospital	550/712 (77.3)	13/20 (65.0)	0.28
Recipient of vaccine			
A(H1N1)pdm09 vaccine§	533/699 (76.3)	2/20 (10.0)	<0.001
Seasonal influenza vaccine 2009–10	258/712 (36.2)	9/20 (45.0)	0.42
Seasonal influenza vaccine 2008–09	470/696 (67.5)	13/20 (65.0)	0.81
Underlying health conditions			
Asthma	67/702 (9.5)	2/20 (10.0)	1.00
Diabetes mellitus	26/700 (3.7)	1/20 (5.0)	0.54
Allergy to airborne irritants	284/646 (44.0)	11/18 (61.1)	0.15
Current smoker	63/704 (9.0)	3/20 (15.0)	0.42
Potential exposure conditions			
Hand-to-face habits**¶**	337/705 (47.8)	14/20 (70.0)	0.05
Wearing of prescription eyeglasses	493/709 (69.5)	12/20 (60.0)	0.36
Reusable water bottle use >1×/ wk	307/705 (43.6)	6/20 (30.0)	0.23
Public transit >8 trips per week	236/706 (33.4)	5/20 (25.0)	0.43
Group gathering attendance >1×	655/710 (92.3)	19/20 (95.0)	1.00
Face-to-face contacts/d, median (IQR)	10 (5, 20)	15 (8, 20)	0.53
Household crowding index >1#	233/697 (33.4)	10/20 (50.0)	0.12
Children in workplace	68/705 (9.7)	3/20 (15.0)	0.43
Child <5 y in household	83/712 (11.7)	4/20 (20.0)	0.28
Child <18 y in household	258/712 (36.2)	13/20 (65.0)	0.009
Child in household attends day care	87/699 (12.5)	6/20 (30.0)	0.03

*Data are no./total (%) unless otherwise specified. A(H1N1)pdm09, pandemic influenza A(H1N1) 2009 virus; IQR, interquartile range. †Participants who did not report any illness or whose nasal swab samples tested negative for influenza. ‡All participants who tested positive were symptomatic. §Participants who had acquired A(H1N1)pdm09 <7 d after vaccination were considered unprotected ¶Defined as biting one’s nails or cuticles or habitually putting one’s fingers in his or her mouth or nose. #Household crowding index is defined as number of persons per household divided by the number of bedrooms.

After adjusting for A(H1N1)pdm09 vaccination history and community influenza activity, we found no difference in the risk for influenza infection between persons working in an acute care hospital (HCWs) and other healthy adults (non-HCWs) (Table 3). Rather, contact with a family member with an ARI in the previous week was the main risk factor for symptomatic influenza infection, irrespective of the method of adjusting for changing risk over time. In general, quasi-likelihood under the independence model criterion statistics were lower in models adjusting for weekly percentage of specimens yielding influenza than in those adjusting for weeks of peak influenza activity (results not shown). A sensitivity analysis calculating lags of 0 and 14 days (vs. 7 days) from the time of receipt of A(H1N1)pdm09 vaccine did not alter these results.

**Table 3:** Risk factors for symptomatic influenza infection among health care workers in acute care hospitals and non–health care workers in office settings during 2009 pandemic, Toronto, Ontario, Canada*

Risk factor	OR (95% CI), adjusted†	OR (95% CI), multivariable‡
Worker in acute care hospital	0.49 (0.19–1.27)	0.47 (0.17–1.32)
Age, y, per 10 y increase	1.08 (0.76–1.54)	NA
Female sex	1.03 (0.30–3.56)	NA
Recipient of A(H1N1)pdm09 vaccine§	0.28 (0.03–2.28)¶	0.34 (0.04–2.85)
Weekly percentage of specimens yielding influenza per 5% increase	1.49 (1.28–1.73)#	1.36 (1.13–1.63)
Potential exposure conditions		
Hand-to-face habits**	3.09 (1.12–8.52)	NA
Child <18 y in household	3.13 (1.21–8.07)	NA
Contact with family member with ARI in prior wk	5.51 (1.81–16.76)	6.89 (2.17–21.84)
Contact with co-worker with ARI in prior wk	0.77 (0.10–6.16)	NA
Household crowding index >1††	1.99 (0.79–5.05)	NA
Public transit >8 trips per wk	0.62 (0.22–1.76)	NA

*Constant and time-dependent risk factors for symptomatic influenza infection (positive nasal swab specimen) in 732 primary contacts of the Influenza Cohort Study followed during June 2009–April 2010, Toronto, Ontario, Canada. OR, odds ratio; NA, not applicable; A(H1N1)pdm09: pandemic influenza A(H1N1) 2009 virus; ARI: acute respiratory illness. †Adjusted for receipt of A(H1N1)pdm09 vaccine and weekly percentage of specimens yielding influenza. ‡Multivariable model including all variables with ORs listed below. §Participants who had acquired A(H1N1)pdm09 <7 d after vaccination were considered unprotected. ¶Adjusted for weekly percentage of specimens yielding influenza only. #Unadjusted. **Defined as biting one’s nails or cuticles, habitually putting one’s fingers in his or her mouth or nose. ††Household crowding index is defined as number of persons per household divided by the number of bedrooms.

Analyses restricted to HCWs and including potential occupational risk factors in health care are shown in Table 4. During the study period, 49.6% of HCWs worked in emergency departments, medical inpatient wards, intensive care units, or pediatric wards; 12.9% were present during >1 and 9.4% performed >1 aerosol-generating medical procedure per week. Approximately one quarter (26.5%) of HCWs reported providing direct care for >1 patient per week who had ARI. The analysis of risk factors for infection indicates that, similar to the combined study population, HCWs with symptomatic influenza infection confirmed by positive nasal swab sample were more likely to have children <18 years of age in their households (69.2% of HCWs who tested positive vs. 36.9% who tested negative or were untested; p = 0.02) and less likely to have been vaccinated against A(H1N1)pdm09 >7 days before onset of infection (15.4% vs. 86.3%; p<0.001) (Table 4). Compared with other HCWs, those with symptomatic influenza infection were more likely to be present during aerosol-generating medical procedures >1× per week (38.5% vs. 12.7%; p = 0.02) and reported lower adherence to hand hygiene recommendations (77.5% vs. 95%; p = 0.02). After adjustment for changing risks for influenza infection over time, risk factors for influenza infection among HCWs were: contact with a family member with ARI in the previous week, performing or assisting with aerosol-generating medical procedures, and lower adherence to hand hygiene recommendations (Table 5).

**Table 4:** Characteristics of 563 health care workers in acute care hospitals during 2009 pandemic, Toronto, Ontario, Canada*

Characteristic	Influenza test status		
	No. negative or not ill, n = 550†	No. positive, n = 13‡	p value
Mean age, y (± SD)	42.2 (11.4)	42.5 (10.1)	0.91
Female sex	467/550 (84.9)	11/13 (84.6)	1.00
Occupation			
Nurse	180/539 (33.4)	3/13 (23.1)	0.55
Physician, physiotherapist, respiratory therapist	103/539 (19.1)	5/13 (38.5)	0.15
Other§	256/539 (47.5)	5/13 (38.5)	0.52
Potential exposure conditions			
Received A(H1N1)pdm09 vaccine¶	467/541 (86.3)	2/13 (15.4)	<0.001
Child <18 y in household	203/550 (36.9)	9/13 (69.2)	0.02
Child attending day care in household	74/542 (13.7)	3/13 (23.1)	0.40
Cares for >1 patient with ARI per week	141/539 (26.2)	5/12 (41.7)	0.32
Working in high-risk area#	227/461 (49.2)	7/11 (63.6)	0.35
Present during aerosol-generating medical procedure >1/wk**	66/521 (12.7)	5/13 (38.5)	0.02
Performs aerosol-generating medical procedure >1/wk	49/540 (9.1)	3/13 (23.1)	0.11
Years’ experience, mean (± SD)	13.6 (11.4)	14.0 (9.3)	0.91
% adherence to hand hygiene, median (IQR)	95.0 (80.0–100)	77.5 (60.0–92.5)	0.02
Adherence to facial protection, %, median (IQR)	80 (50–99)	50 (30–75)	0.16
Hours worked per week, no. median (IQR)	40.0 (37.5–45.0)	37.5 (32.0–40.0)	0.22

*Data are no./total (%) unless otherwise specified. A(H1N1)pdm09, pandemic influenza A(H1N1) 2009 virus; ARI, acute respiratory illness; IQR, interquartile range. †Participants who either did not report any illness or whose nasal swab samples tested negative for influenza. ‡All participants who tested positive were symptomatic. §The distribution of other persons working in acute care hospitals was: administrative personnel: 30.4%; patient attendant/health care aide/service assistant: 0.4%; housekeeper/porter/central sterile supply/dispatch: 0.5%; medical imaging technologist/technician: 1.6%; pharmacist/pharmacy technician: 2.0%; ward clerk/unit coordinator: 1.4%; psychologist/social worker: 1.6%; laboratory technologist/technician: 4.7%; nutritionist/other food service staff: 1.1%; other: 3.4%. ¶Participants who had acquired A(H1N1)pdm09 <7 d of vaccination were considered unprotected. #Emergency room, medical inpatient ward, intensive care unit, or pediatric ward. **Aerosol-generating medical procedures are defined as any one of: administration of nebulized therapy or humidified oxygen at >40%, use of bag-valve mask, manual ventilation, non-invasive ventilation, open airway suctioning, bronchoscopy or other upper airway endoscopy, tracheostomy, endotracheal intubation, cardiopulmonary resuscitation, oscillatory ventilation, any procedure performed that involves manipulation of open ventilator tubing in a mechanically ventilated patient, sputum induction or other deliberate induction of coughing.

**Table 5:** Risk factors for symptomatic influenza infection in health care workers in acute care hospitals during 2009 pandemic, Toronto, Ontario, Canada*

Risk factor	OR (95% CI), adjusted†	OR (95% CI), multivariable‡	
Age, y, per 10 y increase	0.99 (0.63–1.56)		NA
Sex, F	1.79 (0.23–14.04)		NA
Potential exposure conditions			
Receipt of A(H1N1)pdm09 vaccine§	0.49 (0.07–3.67)¶		0.40 (0.04–3.99)
Weekly specimens yielding influenza, %, per 5% increase	1.56 (1.29–1.88) #		1.43 (1.17–1.73)
Child <18 y in household	3.33 (1.00–11.05)		NA
Contact with family member with ARI in prior week	7.26 (2.15–24.54)		7.86 (2.20–28.04)
Contact with co-worker with ARI in prior week	1.40 (0.16–12.40)		NA
Cared for patient with ARI in prior week	1.50 (0.44–5.14)		NA
Adherence to hand hygiene recommendations, per 10% increase	0.84 (0.73–0.98)		0.86 (0.74–0.99)
Adherence to facial protection recommendations, per 10% increase	0.92 (0.79–1.07)		NA
No. AGMP performed or assisted during previous week, per 10 procedures increase**	2.29 (1.26–4.17)		1.95 (1.10–3.48)

*Generalized estimating equation logistic regression analysis of constant and time-varying risk factors for influenza infection in 563 health care workers in acute care hospitals, Influenza Cohort Study, followed during June 2009–April 2010, OR, odds ratio; A(H1N1)pdm09: pandemic influenza A(H1N1) 2009 virus; NA, not applicable; ARI: acute respiratory illness; AGMP: aerosol-generating medical procedures. †Adjusted for receipt of A(H1N1)pdm09 vaccine and weekly percentage of specimens yielding influenza. ‡Multivariable model including all variables with ORs listed below. §Participants who had acquired A(H1N1)pdm09 <7 d after vaccination were considered unprotected. ¶Adjusted for weekly percentage of specimens yielding influenza only. #Undadjusted. **AGMP are defined as any one of the following: administration of nebulized therapy or humidified oxygen at >40%, use of bag-valve mask, manual ventilation, noninvasive ventilation, open airway suctioning, bronchoscopy or other upper airway endoscopy, tracheostomy, endotracheal intubation, cardiopulmonary resuscitation, oscillatory ventilation, any procedure performed that involves manipulation of open ventilator tubing in a mechanically ventilated patient, sputum induction or other deliberate induction of coughing; OR for being in the same room during AGMP (>1/week) 6.63 (95% CI 2.05–21.41); OR for participants performing AGMP (>1/week) 4.21 (1.12–15.76).

### HAI Antibody Assays

Among the combined study population, 450 (61.5%) of 732 participants provided pre- and post-influenza season blood samples. Among those, 3.6% had protective HAI titers against A(H1N1)pdm09 at baseline. There was no association with workplace and baseline HAI titers. Of the 142 (31.6%) participants who tested positive after enrollment, 137 (96.5%) had received the A(H1N1)pdm09 vaccine, 2 (1.4%) submitted a nasal swab that tested positive by PCR, and 3 (2.2%) did not submit a swab for testing or report an ARI (consistent with asymptomatic infection).

Analysis of data collected during the period after vaccine became available for unvaccinated participants without known previous A(H1N1)pdm09 infection showed that 8 (16.3%) of 49 HCW and 3 (5.3%) of 57 non-HCWs seroconverted or had a positive mid-turbinate nasal swab sample. Although persons working in an acute care hospital were 3.1× as likely as other working adults to be infected with influenza, the results were not significant in this small unvaccinated group (95% CI 0.9–11.1). Influenza among unvaccinated participants was not associated with age, sex, or any of the other characteristics listed in Table 1.

## Discussion

In this prospective cohort study conducted in Canada during the 2009 influenza A(H1N1) pandemic, we found no association between working in an acute care hospital and risk for influenza infection. Our findings are similar to those of Williams et al., who assessed serologically confirmed influenza during the 2007–08 influenza season in Berlin, Germany (*14*). They found no association between HCW status and influenza but demonstrated that the presence of children in the household and ownership of a car among participants with no children in the household were risk factors, whereas receipt of seasonal influenza vaccine was found to be protective. Similarly, Marshall et al. found no overall difference in influenza infection rates between hospital workers who did and did not have patient contact during the 2009 pandemic in Australia, but the authors identified exposure to children as a risk for influenza (*15*).

The results of this cohort study also add insight into occupational risk factors for influenza among persons who work in acute care hospitals. In contrast to a finding by Kawana et al. (*16*), neither our study nor those of Marshall et al. and Seto et al. detected an increased risk for influenza among workers who had direct patient care responsibilities (*17*). However, Marshall et al. indicated that working in an intensive care unit of a hospital was a risk factor for influenza, and wearing gloves while caring for patients who were on droplet precaution was protective. These findings are similar to ours in that exposure to aerosol-generating medical procedures, which are most often performed in intensive care units, was a risk factor for influenza, and adherence to hand hygiene, which may have an effect similar to appropriate glove use, was protective. Although collinearity of both putative risk and protective factors may continue to make it difficult to accurately identify risk factors for acquisition of influenza in health care settings, our data highlight the role of hand hygiene in the control of influenza infection (*18*), and of protective equipment use by persons who perform or assist with aerosol-generating medical procedures. 

The mode of transmission of influenza remains a matter of ongoing debate. Although most experts believe that droplet and aerosol transmission are the most common modes of spread of influenza, our finding and that of Marshall et al. (*15*), as well as the evidence from the elementary school–based study by Talaat et al. that increasing hand hygiene adherence reduces the risk for infection with influenza, suggest that transmission by direct or indirect contact contributes substantially to influenza transmission (*18*). Appropriate hand hygiene practice should continue to be recommended to prevent influenza transmission.

Pandemic influenza vaccine became available in Canada at the peak of the second wave of the pandemic. This complicated our analysis in that the risk for influenza infection depended on differing times of receipt of influenza vaccine and on timing of the pandemic waves. We addressed these issues by using multivariable generalized estimating equation logistic regression for the analysis, which facilitated adjustment for timing of receipt of vaccine, and we accounted for the dynamics of the pandemic waves by incorporating weekly percentages of laboratory specimens that tested positive for influenza virus. We believe that our results are robust because 2 different approaches to adjust for changing risk over time led to the same results. Nevertheless, whether the relative percentage of positive specimens reflects the relative number of influenza cases in the community remains a matter of debate.

Our study has several limitations. It has a lack of power related to the small number of cases of symptomatic influenza during the second wave of the pandemic in this population of working adults. We attempted to minimize selection bias by using broad inclusion and limited exclusion criteria; nevertheless, the possibility of having access to rapid diagnosis and treatment during the second pandemic wave might have resulted in biased enrollment of participants who had a higher self-perceived risk for influenza infection, and perception of risk might differ between persons working in acute care hospitals and persons working in nonclinical settings. Similarly, generalizability may be hampered because participants in studies of influenza could differ from others in their attitudes toward vaccine acceptance and infection prevention practices. We tried to reduce the possibility of measurement bias in nasal swab collection by having a broad interpretation of respiratory illness because the interpretation of more detailed criteria for signs or symptoms of influenza infection (e.g., influenza-like illness) might differ between HCWs and non-HCWs, but differences might have remained. Although the self-collection of swab specimens occurred over 1–4 days after illness onset, it is unlikely that any cases would have been missed because previous studies have shown that A(H1N1)pdm09 remains readily detectable within this period (*19*–*21*). The study encompasses a selective sample of persons working in a limited number of acute care hospitals and other working adults with Internet access in a single geographic area during the 2009 influenza A(H1N1) pandemic. Although we deliberately selected controls likely to be at low risk for occupational exposure to influenza (e.g., not working in an occupation exposed to numerous children) in an effort not to miss an effect of the health care work environment, unmeasured biases in our control selection could have been present. In addition, our results may not be generalizable to seasonal influenza or to geopolitical areas where infection control practices in hospitals are different.

The yield of self- or parent-collected nasal swab specimens has been shown to be comparable to health care provider–collected nasopharyngeal aspirates from children and adults (*22*–*24*), but whether the yield of self-collected nasal swabs differs between HCWs and non-HCWs has not been assessed. There is evidence that microneutralization of antibody assays may demonstrate a greater sensitivity than HAI (*25*); as a result, we may have missed seroconversion by using the latter. Further seroconversions might have been missed by the delay between the first (upon enrollment) and the second (April or May 2010) blood sampling caused by declining antibody titers over time. Recall bias might have played a role in that ill participants might have reported risk factors such as contact with sick people in the previous week more accurately than people who did not develop an illness. Finally, participating in the study may have reinforced awareness of the risk for influenza infection and thus may have raised adherence to protective measures.

We did not identify an increase in the risk for influenza among workers in acute care hospitals compared to office-based workers during the 2009 pandemic. However, our findings are limited by lack of power. Within an HCW group, we were able to identify activities that could help focus prevention. Increasing efforts to improve hand hygiene and the use of protective equipment during aerosol-generating medical procedures would further reduce the risk for influenza infection among HCWs.

Technical Appendix Selection and recruitment of non–health care workers enrolled in Influenza Cohort Study, Toronto, Ontario, Canada

## References

[1] Salgado CD, Farr BM, Hall KK, Hayden FG. Influenza in the acute hospital setting. Lancet Infect Dis. 2002;2:145–55 and. 10.1016/S1473-3099(02)00221-911944184

[2] Stott DJ, Kerr G, Carman WF. Nosocomial transmission of influenza. Occup Med (Lond). 2002;52:249–53 and. 10.1093/occmed/52.5.24912181372

[3] Voirin N, Barret B, Metzger MH, Vanhems P. Hospital-acquired influenza: a synthesis using the Outbreak Reports and Intervention Studies of Nosocomial Infection (ORION) statement. J Hosp Infect. 2009;71:1–14 and. 10.1016/j.jhin.2008.08.01318952319

[4] Pearson ML, Bridges CB, Harper SA. Influenza vaccination of health-care personnel: recommendations of the Healthcare Infection Control Practices Advisory Committee (HICPAC) and the Advisory Committee on Immunization Practices (ACIP). MMWR Recomm Rep. 2006;55(RR-2):1–16 .16498385

[5] Talbot TR, Bradley SE, Cosgrove SE, Ruef C, Siegel JD, Weber DJ. Influenza vaccination of healthcare workers and vaccine allocation for healthcare workers during vaccine shortages. Infect Control Hosp Epidemiol. 2005;26:882–90 and. 10.1086/50251216320984

[6] Weber DJ, Rutala WA, Schaffner W. Lessons learned: protection of healthcare workers from infectious disease risks. Crit Care Med. 2010;38(Suppl):S306–14 and. 10.1097/CCM.0b013e3181e69ebd20647788

[7] Elder AG, O'Donnell B, McCruden EA, Symington IS, Carman WF. Incidence and recall of influenza in a cohort of Glasgow healthcare workers during the 1993–4 epidemic: results of serum testing and questionnaire. BMJ. 1996;313:1241–2 and. 10.1136/bmj.313.7067.12418939114PMC2352563

[8] Public Health Agency of Canada. Guidance—H1N1 flu virus [cited 2011 Oct 31]. http://www.phac-aspc.gc.ca/alert-alerte/h1n1/guidance_lignesdirectrices-eng.php#a4

[9] Ontario Ministry of Health and Long-Term Care, Provincial Infectious Diseases Advisory Committee. Best practices for hand hygiene in all health care settings. 2009;37–44 [cited 2011 Oct 31]. http://www.oahpp.ca/resources/documents/pidac/Best%20Practices%20for%20Hand%20Hygiene%20(2009-01).pdf

[10] World Health Organization. WHO manual on animal influenza diagnosis and surveillance. Geneva: The Organization; 2002.

[11] Beyer WE, Palache AM, Luchters G, Nauta J, Osterhaus AD. Seroprotection rate, mean fold increase, seroconversion rate: which parameter adequately expresses seroresponse to influenza vaccination? Virus Res. 2004;103:125–32 and. 10.1016/j.virusres.2004.02.02415163500

[12] Wyatt KN, Ryan GJ, Sheerin KA. Reduced-dose influenza vaccine. Ann Pharmacother. 2006;40:1635–9 and. 10.1345/aph.1G64516835314

[13] Harrell FE. Overview of maximum likelihood estimation. In: Regression modeling strategies with applications to linear models, logistic regression, and survival models. New York: Springer; 2001. p. 179–214.

[14] Williams CJ, Schweiger B, Diner G, Gerlach F, Haaman F, Krause G, Seasonal influenza risk in hospital healthcare workers is more strongly associated with household than occupational exposures: results from a prospective cohort study in Berlin, Germany, 2006/07. BMC Infect Dis. 2010;10:8 and. 10.1186/1471-2334-10-820067628PMC2836320

[15] Marshall C, Kelso A, McBryde E, Barr IG, Eisen DP, Sasadeusz J, Pandemic (H1N1) 2009 risk for frontline health care workers. Emerg Infect Dis. 2011;17:1000–6 and. 10.3201/eid1706.10103021749760PMC3358191

[16] Kawana A, Teruya K, Kirikae T, Sekiguchi J, Kato Y, Kuroda E, “Syndromic surveillance within a hospital” for the early detection of a nosocomial outbreak of acute respiratory infection. Jpn J Infect Dis. 2006;59:377–9 .17186956

[17] Seto WH, Cowling BJ, Lam HS, Ching PT, To ML, Pittet D. Clinical and nonclinical health care workers faced a similar risk of acquiring 2009 pandemic H1N1 infection. Clin Infect Dis. 2011;53:280–3 and. 10.1093/cid/cir37521765076PMC3202320

[18] Talaat M, Afifi S, Dueger E, El-Ashry N, Marfin A, Kandeel A, Effects of hand hygiene campaigns on incidence of laboratory-confirmed influenza and absenteeism in schoolchildren, Cairo, Egypt. Emerg Infect Dis. 2011;17:619–25 and. 10.3201/eid1704.10135321470450PMC3377412

[19] Esposito S, Daleno C, Baldanti F, Scala A, Campanini G, Taroni F, Viral shedding in children infected by pandemic A/H1N1/2009 influenza virus. Virol J. 2011;8:349 and. 10.1186/1743-422X-8-34921752272PMC3150308

[20] Lee CK, Lee HK, Loh TP, Lai FY, Tambyah PA, Chiu L, Comparison of pandemic (H1N1) 2009 and seasonal influenza viral loads, Singapore. Emerg Infect Dis. 2011;17:287–91 and. 10.3201/eid1702.10028221291608PMC3204747

[21] Meschi S, Selleri M, Lalle E, Bordi L, Valli MB, Ferraro F, Duration of viral shedding in hospitalized patients infected with pandemic H1N1. BMC Infect Dis. 2011;11:140 and. 10.1186/1471-2334-11-14021605362PMC3123568

[22] Heikkinen T, Salmi AA, Ruuskanen O. Comparative study of nasopharyngeal aspirate and nasal swab specimens for detection of influenza. BMJ. 2001;322:138 and. 10.1136/bmj.322.7279.13811159569PMC26590

[23] Larios OE, Coleman BL, Drews SJ, Mazzulli T, Borgundvaag B, Green K, Self-collected mid-turbinate swabs for the detection of respiratory viruses in adults with acute respiratory illnesses. PLoS ONE. 2011;6:e21335 and. 10.1371/journal.pone.002133521731708PMC3121745

[24] Smieja M, Castriciano S, Carruthers S, So G, Chong S, Luinstra K, Development and evaluation of a flocked nasal midturbinate swab for self-collection in respiratory virus infection diagnostic testing. J Clin Microbiol. 2010;48:3340–2 and. 10.1128/JCM.02235-0920610685PMC2937679

[25] Papenburg J, Baz M, Hamelin ME, Rheaume C, Carbonneau J, Ouakki M, Evaluation of serological diagnostic methods for the 2009 pandemic influenza A (H1N1) virus. Clin Vaccine Immunol. 2011;18:520–2 and. 10.1128/CVI.00449-1021228145PMC3067392

# Research article: Risk Factors for Influenza among Health Care Workers during 2009 Pandemic, Toronto, Ontario, Canada

**Authors:** Stefan P. Kuster, Brenda L. Coleman, Janet Raboud, Shelly McNeil, Gaston De Serres, Jonathan Gubbay, Todd Hatchette, Kevin C. Katz, Mark Loeb, Donald Low, Tony Mazzulli, Andrew Simor, Allison J. McGeer, on behalf of the Working Adult Influenza Cohort Study Group

**Affiliations:** Mount Sinai Hospital, Toronto, Ontario, Canada (S.P. Kuster, B.L. Coleman, D. Low, T. Mazzulli, A.J. McGeer);; University of Toronto, Toronto (B.L. Coleman, J. Raboud, K.C. Katz, D. Low, T. Mazzulli, A. Simor, A.J. McGeer);; Dalhousie University, Halifax, Nova Scotia, Canada (S. McNeil, T. Hatchette);; Laval University Québec, Québec City, Québec, Canada (G. De Serres); Ontario Agency for Health Protection and Promotion, Toronto (J. Gubbay, D. Low);; North York General Hospital, Toronto (K.C. Katz);; McMaster University, Hamilton, Ontario, Canada (M. Loeb);; Sunnybrook Health Sciences Centre, Toronto (A. Simor).

## Medscape CME Quiz

## Earning CME Credit

To obtain credit, you should first read the journal article. After reading the article, you should be able to answer the following, related, multiple-choice questions. To complete the questions (with a minimum 70% passing score) and earn continuing medical education (CME) credit, please go to www.medscape.org/journal/eid. Credit cannot be obtained for tests completed on paper, although you may use the worksheet below to keep a record of your answers. You must be a registered user on Medscape.org. If you are not registered on Medscape.org, please click on the New Users: Free Registration link on the left hand side of the website to register. Only one answer is correct for each question. Once you successfully answer all post-test questions you will be able to view and/or print your certificate. For questions regarding the content of this activity, contact the accredited provider, CME@medscape.net. For technical assistance, contact CME@webmd.net. American Medical Association’s Physician’s Recognition Award (AMA PRA) credits are accepted in the US as evidence of participation in CME activities. For further information on this award, please refer to http://www.ama-assn.org/ama/pub/category/2922.html. The AMA has determined that physicians not licensed in the US who participate in this CME activity are eligible for *AMA PRA Category 1 Credits*™. Through agreements that the AMA has made with agencies in some countries, AMA PRA credit may be acceptable as evidence of participation in CME activities. If you are not licensed in the US, please complete the questions online, print the certificate and present it to your national medical association for review.

## Article Title:  Risk Factors for Influenza among Health Care Workers during 2009 Pandemic, Toronto, Ontario, Canada

## CME Questions

**1. A 40-year-old nurse at your local hospital presents to your office for a physical examination. She reports a one-day history of fever. That morning, you received a notice that we are currently in the first wave of a pH1N1 pandemic. This is defined as periods for which the weekly proportion of respiratory specimens which yielded pH1N1 was greater than:**

A. 1%

B. 5%

C. 10%

D. 25%

**2. You suspect the patient in question 1 may have pH1N1 when she reported the following symptoms:**

A. Current urinary tract infection symptoms

B. Sore throat and cough

C. Runny nose and headache

D. Answers B and C

**3. The patient in question 1 asks you if her risk of influenza is higher due to her occupation. You inform her that:**

A. Her risk is higher compared to non-healthcare workers (HCWs)

B. Her risk is lower compared to non-HCWs

C. There is no difference in risk compared to non-HCWs

D. None of the above is correct

**4. What other risk factors should you inquire about the patient in question 1 that would increase her risk of influenza?**

A. Performing aerosol-generating medical procedure

B. Recent pH1N1 vaccination

C. High adherence to hand hygiene recommendations

D. Working in the pediatric ward

## Activity Evaluation

**1:** The activity supported the learning objectives.

Strongly Disagree				Strongly Agree
1	2	3	4	5
**2. The material was organized clearly for learning to occur.**				
Strongly Disagree				Strongly Agree
1	2	3	4	5
**3. The content learned from this activity will impact my practice.**				
Strongly Disagree				Strongly Agree
1	2	3	4	5
**4. The activity was presented objectively and free of commercial bias.**				
Strongly Disagree				Strongly Agree
1	2	3	4	5
